# All *trans* retinoic acid as a host-directed immunotherapy for tuberculosis

**DOI:** 10.1016/j.crimmu.2022.03.003

**Published:** 2022-03-30

**Authors:** Ahmad Z. Bahlool, Conor Grant, Sally-Ann Cryan, Joseph Keane, Mary P. O'Sullivan

**Affiliations:** a School of Pharmacy and Biomolecular Sciences (PBS), Royal College of Surgeons in Ireland (RCSI), 123 St Stephens Green, Dublin 2, Ireland; b Tissue Engineering Research Group (TERG), Royal College of Surgeons in Ireland (RCSI), 123 St Stephens Green, Dublin 2, Ireland; c Department of Clinical Medicine, Trinity Translational Medicine Institute, St. James's Hospital, Trinity College Dublin, The University of Dublin, Dublin 8, Ireland; d SFI Advanced Materials and Bioengineering Research (AMBER) Centre, RCSI & TCD, Dublin, Ireland; e SFI Centre for Research in Medical Devices (CURAM), RCSI, Dublin and National University of Ireland, Galway, Ireland

**Keywords:** Tuberculosis (TB), Host-directed therapy (HDT), Retinoic acid (ATRA), Macrophage

## Abstract

Tuberculosis (TB) is the top bacterial infectious disease killer and one of the top ten causes of death worldwide. The emergence of strains of multiple drug-resistant tuberculosis (MDR-TB) has pushed our available stock of anti-TB agents to the limit of effectiveness. This has increased the urgent need to develop novel treatment strategies using currently available resources. An adjunctive, host-directed therapy (HDT) designed to act on the host, instead of the bacteria, by boosting the host immune response through activation of intracellular pathways could be the answer. The integration of multidisciplinary approaches of repurposing currently FDA-approved drugs, with a targeted drug-delivery platform is a very promising option to reduce the long timeline associated with the approval of new drugs - time that cannot be afforded given the current levels of morbidity and mortality associated with TB infection. The deficiency of vitamin A has been reported to be highly associated with the increased susceptibility of TB. All *trans* retinoic acid (ATRA), the active metabolite of vitamin A, has proven to be very efficacious against TB both *in vitro* and *in vivo*. In this review, we discuss and summarise the importance of vitamin A metabolites in the fight against TB and what is known regarding the molecular mechanisms of ATRA as a host-directed therapy for TB including its effect on macrophages cytokine profile and cellular pathways. Furthermore, we focus on the issues behind why previous clinical trials with vitamin A supplementation have failed, and how these issues might be overcome.

## Highlights

• Tuberculosis deaths and resistance are increasing – novel therapies are needed.
• Vitamin A deficiency is a strong risk factor for active tuberculosis in contacts.
• All *Trans* Retinoic Acid is a promising host-directed therapy for tuberculosis.
• It has pleiotropic effects on macrophages & other immune cells *in vitro* and *in vivo*.
• Inhaled rather than systemic All *Trans* Retinoic Acid therapy may be most effective.

## 1 Tuberculosis

Ending Tuberculosis (TB) by 2030 was listed in the UN sustainable development goals as it kills more people globally than any other infection (Health – United Nations S, 2021). The COVID-19 pandemic is significantly impacting TB case-finding, management and access to treatment, leading to a drop in diagnosis and an increase in deaths compared with previous years. According to the latest 2021 World Health Organization (WHO) global TB report, 5.8 million people newly diagnosed with TB in 2020. In the same year 1.3 million HIV negative and 214,000 HIV positive people died from TB (Global tuberculosis repor, 2021). There is only one approved TB vaccine, the Bacille Calmette-Guérin (BCG) vaccine. A meta-analysis estimated that the BCG's duration of protection in pediatric populations is generally up to 10 years (Abubakar et al., 2013). The efficacy of the BCG vaccine against TB in adults is highly variable, ranging from 0 to 80% in different geographic locations (Mangtani et al., 2014). This sounds the alarm globally for the need of new treatment strategies to achieve the UN and WHO goals by 2030.

*Mycobacterium tuberculosis* (Mtb), the causative microorganism of TB discovered by Robert Koch, is transmitted via inhalation of respiratory droplets containing Mtb bacilli, which are then phagocytosed by alveolar macrophages (AM) as well as other phagocytes. Mtb blocks phagolysosomal maturation, avoids destruction, and remains dormant inside the macrophage (Kiran et al., 2016; Pai et al., 2016). The host-mediated Th1 response involves granuloma formation which often results in containment of infection in healthy individuals, but not eradication of the infection – so-called latent TB infection (LTBI) (Saunders et al., 1999). TB granuloma has been studied in the lungs of TB patients (Long et al., 1998) explant tissues (Tsai et al., 2006) animal models (Via et al., 2008) and *in vitro* models (Puissegur et al., 2004). These studies have found that Mtb-infected monocytes undergo differentiation into several cell types including mononucleated giant cells (MNGCs), macrophages and epithelioid cells. One the other hand, Mtb can continue to multiply causing cell rupture and bacterial dissemination to other parts of the lungs and body, leading to active TB disease which is considered as the contagious state (active TB) (Esmail et al., 2014).

Standard treatment for TB includes an intensive phase consisting of two months of pyrazinamide (PZA), isoniazid (INH), rifampin (RIF), and ethambutol (EMB) followed by a continuation phase of 4 months of INH and RIF (Nahid et al., 2016). However, poor patient adherence can be an issue due to the long duration of treatment and the drugs’ side effects (Cohn, 2000), which can contribute to the rise of MDR strains. MDR-TB is defined as Mtb resistant to at least isoniazid and rifampicin. Treatment regimens of MDR-TB include the use of at least 5 drugs for 5–7 months in the intensive phase and 4 drugs in the continuation phase, with a total treatment duration of 15–21 months (Nahid et al., 2019). Extensive drug resistant TB (XDR-TB) is defined as resistance to rifampicin, isoniazid and the second line fluoroquinolones and aminoglycosides (Pai et al., 2016). Thus, new TB treatment strategies should be considered to overcome the issue of drug resistance, increase the options available for these difficult-to-treat patients and improve regimen tolerance - thereby improving treatment adherence.

## 2 Host directed therapy (HDT) for TB

Despite an estimated one quarter of the world's population having LTBI, only 10 million people had active TB infections in 2019 (R, 2020). Thus, the host immune system is capable of successfully controlling the infection in the majority of those infected and it should not be underestimated. The concept of HDTs describes therapeutic strategies that target host immune responses to augment beneficial features of fighting the bacteria and reduce harmful effects of tissue damage (O'Connor et al., 2016). The most attractive features of this treatment strategy are the lower potential of developing drug resistance by Mtb, the possibility to use a HDT as a vaccine adjuvant or as a prophylactic for close contacts, and HDT's potential to improve the overall pathology of TB disease by limiting excessive inflammation.

Additionally, the repurposing of licensed medications as HDT is considered as a faster approach for market access and lowering industrial development-related costs (Fatima et al., 2021; Oprea et al., 2011). In parallel with the interest in drug repurposing and HDT, there has been great interest in new drug delivery approaches to facilitate drug targeting to the site of primary Mtb infection in the lungs and alveolar macrophages (Batalha et al., 2019; Kalombo et al., 2019; Dua et al., 2018). A new chemical entity (NCE) can take up to 20 years to gain market authorisation under the current regulations. There has been a renewed focus on the benefits of drug repurposing as part of the global efforts to tackle COVID-19. The repurposing of approved drugs could be an answer to reduce the long drug development timelines that the TB community cannot afford.

HDT for Mtb infection includes a number of pathways targeted by a broad range of compounds including but not limited to; autophagy inducers such as rapamycin and the antihyperglycemic agent metformin (Coleman et al., 2018; Gutierrez et al., 2004; Singhal et al., 2014; Vashisht and Brahmachari, 2015), metabolic regulators such as the lipid lowering statins (Parihar et al., 2014), cytokine modulators (Murray, 1994), corticosteroids (Critchley et al., 2013) and protein kinase inhibitors such as Imatinib (Napier et al., 2011). Clinical practice guidelines recommend multi-drug combination regimens to effectively treat TB, reduce the risk of relapse and reduce the development of resistance (Nahid et al., 2019). Therefore, any new HDT formulation could be considered as an adjunctive treatment to the current regimens.

The host immune response to Mtb infection is very complex and many host functions that are important in the early stages are considered detrimental at later stages (Roca and Ramakrishnan, 2013). Genetic variation is also an important factor to be considered in the era of personalized medicine. These variations in host immunity may significantly impact the response to HDT to a greater extent than classical antimicrobials (Tobin et al., 2012; Olaru et al., 2016). Thus, it is important to have a comprehensive understanding of the mechanisms of action of HDTs and their interaction with the host immune system in order to properly implement them in the current treatment regimens, and prevent any underestimation of their clinical effects.

### 2.1 Vitamins as HDT

It is well-known that TB is a disease of poverty that occurs mainly in low- and middle-income countries (R, 2020). Epidemiological evidence shows that nutritional status and body mass index affects the host response to TB (Tellez-Navarrete et al., 2021; Lonnroth et al., 2010). Similarly, the nutritional status of the host may be important for the efficacy of any manipulation of the host immune response. Micronutrients are of great importance in the ability of the immune system to fight against microbes. Several promising HDT including vitamins have been studied for their ability to influence the host cell metabolism and gene regulation (Newton et al., 2020; Carlberg, 1999). In patients with Mtb infection, levels of vitamins A, C and E are lower than in healthy individuals, which leads to increased oxidative stress. Administration of these vitamins may reduce oxidative stress and reduce excessive inflammation and thereby support more favourable immune responses (Amaral et al., 2021; Kurutas, 2016). Vitamins B, C, E and A are considered as antioxidants that reduce cell damage caused by free radicals (Mora et al., 2008). Pyridoxine (vitamin B6) is usually prescribed with isoniazid (INH) to reduce the risk of peripheral neuropathy (Snider, 1980). Vitamin E might have a role in TB due to its anti-inflammatory effects which may also reduce tissue damage in TB lungs (Mora et al., 2008).

Vitamin derivatives are cheap, widely available and prescribed for many prophylactic and therapeutic indications. In the pre-antibiotic era, micronutrients in cod liver oil such as vitamin D and vitamin A were administered as anti-infective agents for many conditions including TB (Semba, 1999a). Vitamin D is a fat soluble endogenous vitamin that undergoes metabolism from 7-dehydrocholesterol to 25-dihydroxyvitamin D3, then to 1,25-dihydroxyvitamin D3 (1,25D) and acts on the vitamin D receptor, heterodimerising with the nuclear retinoid X receptor family (RXR) (Mora et al., 2008). Vitamin D is currently licenced for treatment of osteoporosis and psoriasis (Leyssens et al., 2014). Lower vitamin D levels are associated with higher mortality in critically ill patients (Leaf et al., 2015). The antimycobacterial effects of vitamin D have been documented since the 1980s; Mtb infected human macrophages treated with (1,25D) showed slowed bacterial growth (Crowle et al., 1987). It exerts its antimicrobial activity via a 37-amino acid protein, called cathelicidin antimicrobial peptide (CAMP), generated by immune cells (Liu et al., 2007). It also exerts a host-protective effect by modulating the T-helper 1 (Th1) proinflammatory response which might be useful in avoiding excessive inflammation (Mora et al., 2008).

## 3 Vitamin A deficiency as a risk factor for tuberculosis: Epidemiology and causes

### 3.1 Epidemiology of vitamin A deficiency

In developed, high resource settings such as Europe, dietary surveys indicate that few children or adults have a vitamin A intake lower than what is recommended (900 mcg for adult men and 700 mcg for adult women) (Mensink et al., 2013). Unsurprisingly, the sequelae of vitamin A deficiency (VAD), which are rarely seen in these settings, are usually the result of restrictive diets or malabsorption, and are the subject of case reports (Lin et al., 2011; Simkin et al., 2016). It is estimated that 1.1% of all global mortality, and 1 in 5 deaths from diarrheal illness are attributable to VAD. Likewise, 1.5% of global disability adjusted life years have been attributed to VAD. VAD causes xerophthalmia and is the leading cause of acquired blindness in children. Night-blindness affects 2% of African and 0.5% of South East Asian children (Organization, 2009; Sommer and Vyas, 2012; Sherwin et al., 2012).

Vitamin A has a central role in development, growth, cell proliferation, epithelial integrity, and immunity (Wiseman et al., 2017). High-risk populations are those in low resource settings where crop yields and infrastructure are poor, where diets are low in fat and where populations depend on rice, which lacks carotenoids. Within these populations, young children following weaning, and pregnant women are at particularly high risk given their increased requirements (Organization, 2009; Sherwin et al., 2012). Consequently, high-dose vitamin A supplementation is recommended by the World Health Organization for all children aged 6–59 months and pregnant women in settings where vitamin A deficiency is prevalent (Guideline: vitamin A supp, 2021; O recommendations on an, 2021). In fact, meta-analyses evaluating the efficacy of vitamin A supplementation in these children have estimated a relative risk reduction in all-cause mortality of about one quarter (Imdad et al., 2011; Mayo-Wilson et al., 2011). Vitamin A supplementation is also recommended in the treatment of severe acute malnutrition, as it reduces mortality (Organization, 2021).

### 3.2 Vitamin A deficiency in infectious diseases

Plasma retinol levels are lower in patients with infectious disease rather than other disease types (Chau et al., 2000). Whether this is a cause or an effect of infection (or more likely both) is difficult to unpick. As discussed above, supplementation has successfully reduced infection-related mortality in certain populations, which supports the hypothesis that VAD results in immune dysfunction and infection (Imdad et al., 2011; Mayo-Wilson et al., 2011). However, inflammation also reduces plasma retinol and retinol binding protein-4 (RBP4) levels (Larson et al., 2017; Louw et al., 1992). Retinol is excreted in the urine of patients with infections (Alvarez et al., 1995; Stephensen et al., 1994) and certain infections, giardiasis or ascariasis for example, might reduce intestinal absorption of vitamin A (Al-Mekhlafi et al., 2010; de Gier et al., 2014). Also, in the absence of inflammation, low circulating retinol levels only reflect liver vitamin A stores when they are severely depleted, making its interpretation more difficult (Larson et al., 2017).

Notwithstanding, hyporetinolaemia and VAD have been associated with many types of infections: a large, prospective, longitudinal cohort study of Colombian children found significant relationships between plasma retinol levels and reductions in the risks of gastroenteritis, respiratory tract infections, otitis and need for medical attention (Thornton et al., 2014). VAD is associated with an increased severity of measles infection. A Cochrane systematic review found that two doses of vitamin A significantly reduced the mortality of young children with measles (Frieden et al., 1992; Yang et al., 2005). Hyporetinolaemia was not associated with progression of HIV infection in the 1990s, but is an independent predictor of non-response to interferon therapy for Hepatitis C virus infection, unlike vitamin D (Bitetto et al., 2013; Tang et al., 1997). Giardiasis, ascariasis, or infection with any soil-transmitted helminth have repeatedly been found to be associated with VAD (Al-Mekhlafi et al., 2010; de Gier et al., 2014; Suchdev et al., 2014). While retinol supplementation supports host anti-helminthic cytokine responses in children with *Ascaris* infections, routine supplementation following deworming does not appear to reduce reinfection rates or intensity in endemic settings (Long et al., 2006; Al-Mekhlafi et al., 2014). Oh et al., report that VAD was significantly more prevalent in patients with pulmonary nontuberculous mycobacterial disease than healthy controls (Oh et al., 2019).

### 3.3 Vitamin A deficiency in active tuberculosis

Many case-control studies have found an association between TB and low retinol levels or VAD. These studies have a wide geographic spread, and were undertaken in South Korea (Oh et al., 2017), Ethiopia (Keflie et al., 2018), India (Ramachandran et al.), Tanzania (Mugusi et al., 2003) and South Africa (Plit et al., 1998). While our understanding of vitamin A's immunological role and promising *in vitro* results suggest a direction of causality, the systemic inflammatory responses of TB patients complicate the interpretation of these case-control studies as providing strong evidence for VAD causing TB-risk. However, two important prospective cohort studies following high-risk groups over time lend strong support to the theory that hyporetinolaemia is a risk factor for active TB. Aibana et al., followed 6751 HIV-negative household contacts (HHCs) of TB cases in Peru for one year. Even after adjusting for many confounders, the authors found that HHCs with baseline VAD had more than a 10-fold increased risk of developing TB, or 20-fold for those aged between 10 and 19 years. Notably, there was a stepwise increase in TB risk with each vitamin A quartile, even for contacts who were not deficient (Aibana et al., 2017). In addition, Tenforde and colleagues followed HIV positive patients starting antiretroviral therapy (ART) in nine countries. When comparing those who developed active TB within 96 weeks, and after adjusting for confounders, pre-ART VAD was associated with a 5.3 hazard ratio of developing active TB. This risk remained significant even after adjusting for Vitamin D status (Tenforde et al., 2017). Two studies have investigated the association of vitamin A deficiency and severity of TB diseases. In Indonesia, a cross sectional study of 300 smear positive TB patients, found VAD in 64% of patients with severe disease but only 37% of patients with mild disease (Pakasi et al., 2009). However, in Morocco, Qrafli et al., examined 44 smear positive TB cases and did not find an association between plasma retinol and TB disease severity (Qrafli et al., 2017).

In summary, multiple lines of evidence suggest that infection is associated with hyporetinolaemia and that, reciprocally, VAD is associated with an increased risk of several infections affecting the respiratory or gastrointestinal mucosa in children, and an increased risk of acquiring active tuberculosis in adults. Randomised controlled trials that intervene to correct VAD and measure the incidence of active TB are needed to determine if VAD is causally associated with TB and if correction of VAD is a clinically effective prophylactic strategy for TB. These trials could focus on high-risk groups with VAD such as patients starting antiretroviral therapy for HIV, as studied by Tenforde et al. (2017), or household contacts of TB cases, as studied by Aibana et al. (2017). Indeed, supplementation of vitamin A should be trialed irrespective of vitamin A status given the inverse correlation between baseline serum retinol level and risk of active TB even among non-deficient contacts.

## 4 Vitamin A metabolism

### 4.1 Vitamin A absorption & storage

Dietary vitamin A takes the forms of preformed retinoids, retinol or retinyl esters, found in animal food sources and pro-retinoid carotenoids, such as β-carotene found in plant food sources. Vitamin A absorption is negatively correlated with fever (Aklamati et al., 2010). Retinyl esters are converted to retinol in the intestinal lumen and carotenoids are converted to retinol in enterocytes. Retinol is then esterified in the enterocyte by lecithin retinol acyltransferase (LRAT) and secreted in chylomicrons into circulation via the lymphatic system – a process important to host defense in a murine model of TB infection (Blaner et al., 2016; Li et al., 2014; Kono and Arai, 2015; Trasino et al., 2020). Hepatocytes endocytose circulating chylomicron remnants. Retinol-binding protein 4 (RBP4) is synthesised in hepatocytes and binds to retinol in the endoplasmic reticulum, at which point retinol-RBP4 is either shipped to stellate cells for storage or secreted into circulation for transport to target tissues (Kono and Arai, 2015). Almost all of the body's total retinoid is stored in hepatic stellate cells in retinyl ester form (Blaner et al., 2016; Li et al., 2014).

### 4.2 Vitamin A transport

**Fig. 1:**
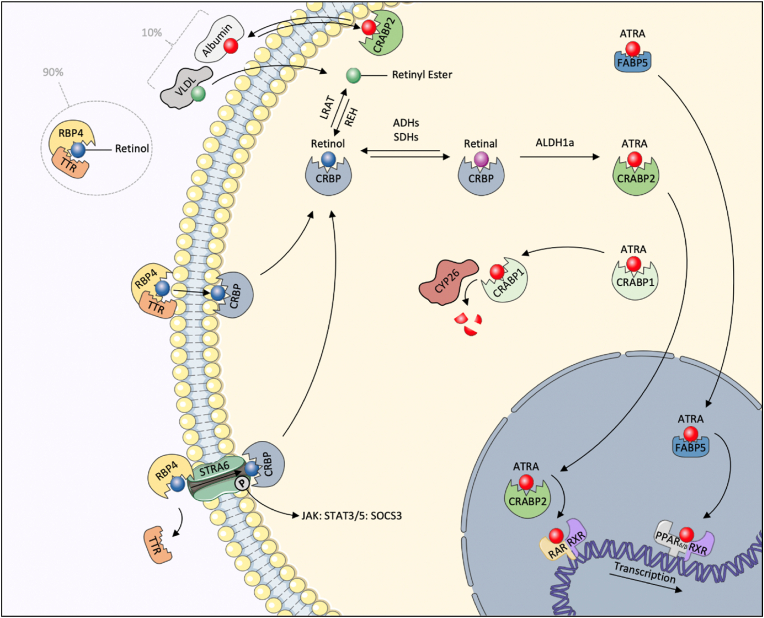
Vitamin A is delivered to target cells in several ways: Retinyl esters (in green) can be delivered within chylomicrons post-prandially or within liver-secreted VLDL. Retinoic acid (in red), can be produced by neighbouring cells and delivered, bound to albumin. However, 90% of all circulating retinoids are in the form of retinol (in blue), bound to RBP4 and TTR. Retinol can diffuse freely across cell membranes or in certain cells, following dissociation of TTR, can also be channelled through the surface receptor STRA6, which triggers a JAK-STAT3/5 signalling cascade. Intracellularly, retinol is bound to Cellular Retinol Binding Protein (CRBP), which also binds retinal (in purple). If not esterified by LRAT, retinol can be reversibly oxidised to retinal by short-chain dehydrogenases or alcohol dehydrogenases. Retinal can then be irreversibly oxidised to retinoic acid by members of the aldehyde dehydrogenase 1a family. ATRA (in red), the predominant isoform of retinoic acid, is bound to Cellular Retinoic Acid Binding Protein 1 or 2 (CRABP1, CRABP2), or Fatty Acid Binding Protein 5 (FABP5). CRABP1 preferentially delivers ATRA to Cytochrome P450 26 (CYP26) for degradation to polar metabolites, whereas CRABP2 delivers ATRA to its primary nuclear receptor, the RAR:RXR heterodimer, to activate transcription of its target genes. If the ratio of FABP5 to CRABP2 is high, ATRA is preferentially delivered to an alternative nuclear receptor, the PPARδ/β:RXR heterodimer. (For interpretation of the references to colour in this figure legend, the reader is referred to the Web version of this article.)

There are several pathways through which retinol is delivered to target tissues, including as dietary retinoids in chylomicrons (before liver processing) or as liver-secreted retinyl esters bound to VLDL (Blaner et al., 2016; Steinhoff et al., 2021). However, nearly all of circulating retinoids are in the form of retinol bound to Retinol Binding Protein 4 (RBP4) and a second protein, transthyretin (TTR) in a 1:1 ratio (Blaner et al., 2016; Li et al., 2014). RBP4 is a 21 kDa lipocalin protein that chaperones hydrophobic retinol in the circulation (Berry et al., 2012a, 2012b) (Fig. 1). RBP4 is required for deployment of liver retinoid stores, and retinol is required for the secretion of RBP4 from hepatocytes (Blaner et al., 2016). Low circulating RBP4 levels can indicate VAD, however only after liver stores are depleted (Li et al., 2014). RBP4 mutations are associated with visual defects, obesity, cardiovascular disease and hypertriglyceridemia (Steinhoff et al., 2021). RBP4 is a negative acute phase response protein that is downregulated in inflammation, such as in the postoperative period (Louw et al., 1992). Retinol and RBP4 were inversely correlated with CRP in Cameroonian women and children, but positively correlated with haemoglobin (Engle-Stone et al., 2011). This complicates the interpretation of retinol or RBP4 levels as indicators of vitamin A sufficiency or deficiency in inflammatory states. Unsurprisingly, RBP4 levels are lower in TB patients (Keicho et al., 2012). A recent proteomic study by Jarsberg et al., found that in African patients, TTR levels improved faster with TB treatment than RBP4, which depends on liver retinoid stores for its secretion. This suggests that vitamin A deficiency, and not just the inflammatory response, causes low RBP4 in this group of TB patients (Jarsberg et al., 2021). In the inflammatory state, another retinol-binding protein may take over the function of RBP4: Serum amyloid A (SAA) was shown to be responsible for transport of retinol to myeloid cells during bacterial infection (Hu et al., 2019; Derebe et al., 2014). SAA delivers retinol to RA-producing intestinal myeloid cells, where it binds to the transmembrane receptor LRP1 (Bang et al., 2021).

Another, unexplored factor that may reduce retinol-RBP4 levels in TB patients is urinary excretion. While only trace amounts are excreted in healthy adults and children, substantial quantities are excreted in patients with fever, pneumonia, sepsis and rotaviral diarrhea (Alvarez et al., 1995; Stephensen et al., 1994). RBP4 levels are also correlated with obesity, hepatic steatosis, triglyceride levels and VLDL-cholesterol levels. In obese patients undergoing bariatric surgery, weight loss was independently associated with a reduction in RBP4 (Broch et al., 2010; Stefan et al., 2007; von Eynatten et al., 2007). While weight loss is emblematic of TB disease, and it is tempting to hypothesise that TB-induced weight loss is another factor reducing circulating retinol-RBP4, these cohorts are quite distinct.

Transthyretin (TTR) is a 55 kDa protein that prevents renal excretion of retinol-RBP4 by binding the complex in circulation (Steinhoff et al., 2021; Berry et al., 2012a). Interestingly, TTR also serves as a transporter of thyroxine and tri-iodothyronine. Like RBP4, TTR is a negative acute phase protein that is suppressed in inflammatory states. Agranoff et al., analysed the serum of 179 culture-confirmed TB cases and 170 controls with other inflammatory or infectious conditions. They found that TTR was one of the most discriminatory proteins between cases and controls (Agranoff et al., 2006).

### 4.3 Vitamin A cell entry

TTR needs to dissociate with the retinol-RBP4 complex before the retinol-RBP4 complex can associate with its cell surface receptor (STRA6). STRA6 binds RBP4, and facilitates the transmembrane channelling of retinol to an intracellular acceptor, the cellular retinol binding protein (CRBP) (Kono and Arai, 2015; Steinhoff et al., 2021). Retinol-RBP4 also phosphorylates STRA6, triggering a signalling cascade that ends in STAT3 and STAT5 activation and SOCS3 signalling (Fig. 1). This retinol-RBP ‘sensing’ is thought to underlie the known link between vitamin A homeostasis and insulin resistance, and represents an effector pathway of vitamin A in addition to those mediated by retinal and retinoic acid (Muenzner et al., 2013; Berry et al., 2011). However, most cellular uptake of retinol from the retinol-RBP4-TTR complex is not through STRA6, and retinol is thought to diffuse freely across the cell membrane (Berry et al., 2011). In fact, while retinol has beneficial anti-TB effects in human monocyte-derived macrophages (MDM), they do not express STRA6 (Coleman et al., 2018; Norseen et al., 2012). As STRA6 is not necessary for cellular entry of retinol, its primary function is thought to be that of ‘sensing’ and signalling (Berry et al., 2011).

### 4.4 Enzymes involved in vitamin A metabolism

While retinol itself is inert, its metabolite retinal is essential for vision and its metabolite retinoic acid exerts many effects by activating nuclear receptors in its target cells. These active metabolites are manufactured in situ in target cells from retinol, or alternatively retinoic acid diffuses from neighbouring cells. Retinoic acid has three isoforms: all-trans retinoic acid (ATRA), 9-cis RA and 13-cis RA, with ATRA being the most abundant and active isoform (Gonçalves et al., 2019). These metabolites are produced in a two-step oxidation reaction in the target tissues. Firstly, retinol is reversibly oxidised to retinal by the widely expressed alcohol dehydrogenases (ADH), and short-chain dehydrogenase/reductases (SDR) when retinol is protein-bound (Blomhoff and Blomhoff, 2006). Secondly, members of the aldehyde dehydrogenase (ALDH) family irreversibly oxidise retinal to ATRA or its isomers. These include ALDH1a1, ALDH1a2 and ALDH1a3 (Fig. 1). While oxidation of retinol to retinal is not tissue restricted, the tissue-specific expression of the ALDH1a enzymes determines which tissues are capable of producing vitamin A's active metabolites (Duester et al., 2003; Isoherranen and Zhong, 2019; Stevison et al., 2015).

There is some evidence suggesting that human macrophages express these enzymes and are capable of producing ATRA. DHRS9, an SDR, is highly expressed in regulatory macrophages that suppress T cell proliferation, and reduced in interferon gamma-stimulated macrophages (Riquelme et al., 2017). In keeping with this, the expression of DHRS9 and ALDH1a2 were significantly lower in caseous TB lung tissue than in unaffected tissue, although the cells expressing these enzymes were not identified (Kim et al., 2019). In a rabbit model of TB infection, ALDH1a2 was significantly reduced in lung tissue following infection (Kim et al., 2019). Similarly, mice infected with *Trichuris muris* had reduced ALDH activity in their intestinal macrophages (Erkelens and Mebius, 2017). ALDH1a2 expression was detected in alternatively activated macrophages in murine liver during *Schistosoma mansoni* infection and IL-4 also induced its expression in peritoneal macrophages *in vivo* and in bone marrow derived macrophages (BMDM) *in vitro* (Broadhurst et al., 2012). Niu et al. reported that resting murine BMDM do not express ALDH1a1, ALDH1a 2 or ALDH1a 3 but rather synthesise ATRA using the enzyme ALDH3b1 (Niu et al., 2016). Human intestinal macrophages express ALDH1a1 and ALDH1a2, but not ALDH1a3 (Sanders et al., 2014; Denning et al., 2011). ALDH1a activity was detected in human alveolar macrophages using a low-specificity Aldefluor assay (Coleman et al., 2013). Overall, there is a paucity of data identifying which vitamin A-metabolising enzymes are expressed in the human macrophage populations.

Blocking the oxidation of retinol to ATRA in human macrophages with the ALDH inhibitor N,N-diethylaminobenzaldehyde (DEAB) undermines their ability to kill Mtb (Coleman et al., 2018). Enhancing the cellular production of ATRA in human macrophages by increasing the expression of these enzymes may benefit the host response to Mtb, acting as a HDT. ALDH1a-family enzyme induction by IL-4, GM-CSF and PPAR-γ agonists has been observed in human monocyte-derived dendritic cells (DCs) (Kim et al., 2019; Agace and Persson, 2012; Gyöngyösi et al., 2013). This approach may be constrained by a negative feedback inhibition of ATRA on ALDH1a1 expression (Ito et al., 2014).

### 4.5 Carrier proteins

Intracellular retinoid-binding proteins chaperone the hydrophobic retinoids in the cytosol. Cellular Retinol-Binding Proteins (CRBP) bind retinol and retinal. Cellular Retinoic Acid-Binding Proteins, ubiquitous CRABP1 and more selectively-expressed CRABP2, bind ATRA (Bushue and Wan, 2010). CRABP2 is expressed in human MDMs (Kreutz et al., 1998). It has been suggested that the expression of these carrier proteins, and not the oxidation enzymes, is the rate-limiting step in the production of ATRA (Niu et al., 2016). In addition, there is evidence to suggest that the relative expression of CRABP1, CRABP2 and fatty acid binding protein 5 (FABP5) may determine the fate of ATRA – whether it is degraded and which nuclear receptors are activated. In summary, CRABP1 may target ATRA for degradation by cytochrome P450 enzymes, CRABP2 delivers ATRA to the Retinoic acid receptor (RAR) nuclear receptor and FABP5 delivers ATRA to the PPAR δ/β nuclear receptor (Al Tanoury et al., 2013; Larange and Cheroutre, 2016; Napoli, 2017). PPAR-γ agonists increase CRABP2 expression, and thus may increase the protective effects resulting from ATRA delivery to the RAR receptor (Gyöngyösi et al., 2013) (Fig. 1).

### 4.6 Degradation by cytochrome P450

ATRA is degraded to polar metabolites by members of the Cytochrome P450 26 family (CYP26A1, CYP26B1 and CYP26C1) (Isoherranen and Zhong, 2019; Guo et al., 2015) (Fig. 1). The expression of these enzymes is strongly increased by ATRA, which may cause treatment-resistance if ATRA is used as a HDT (Chen et al., 2019). CYP26 inhibitors used alone, or in combination with ATRA, are worth considering when exploring ATRA as a HDT for Mtb.

## 5 ATRA as a promising host directed therapy for TB

Vitamin A cannot be synthesised de novo in the body and must be obtained from diet (Palace et al., 1999). All *trans* retinoic acid (ATRA), known as Tretinoin, is the active metabolite of vitamin A. Tretinoin is currently used as treatment for acne and acute promyelocytic leukaemia (APL) (Alizadeh et al., 2014). Our lab has previously reported that human alveolar macrophages display ALDH1a activity and produce ATRA and this induces FoxP3^+^ regulatory T-cells that supress inflammation and thus reduce unwanted tissue damage caused by the immune system (Coleman et al., 2013). Three vitamin A metabolites; ATRA, 13-*cis* retinoic acid and retinyl acetate resulted in dose-dependent inhibition of the growth of several mycobacterium species *in vitro*. ATRA and 13-*cis* retinoic acid were the most effective against Mtb (Greenstein et al., 2012). One of the earliest reports of the role of retinoic acid in TB was in 1989, and showed that RA was bacteriostatic against Mtb in human MDMs if added before infection at physiologic concentration or at pharmacological concentrations after infection (Crowle and Ross, 1989).

Retinoids regulate gene transcription which is mediated by binding to RAR and RXR (Huen and Kim, 2015). Each family of these receptors is associated with three subtypes (alpha, beta, gamma) and each one of them can present in several isoforms (Das et al., 2014). Human RARs can be activated by the ligands all-trans RA, 9-cis RA, 13-cis RA, etretinate and acitretin, while the ligands for human RXRs are only 9-cis RA and bexarotene (Huen and Kim, 2015). RXRs can form homodimers or heterodimers with RAR and other receptors such as vitamin D receptor (VDR), bile acids Farnesoid X Receptor (FXR) and fatty acids peroxisomal proliferator activated receptors (PPAR) (Bushue and Wan, 2010). Therefore, several signalling pathways can be activated. The RAR/RXR heterodimer is mainly responsible for the biological activity of RA and it is believed that most of the RA effects are mediated by this receptor dimer (Lei and De Thé, 2003). The RXR-RAR heterodimers bind to certain retinoic acid responsive elements (RAREs) located in target gene promoters and regulate gene expression (Rochette-Egly and Chambon, 2001; Chatagnon et al., 2015; Rochette-Egly and Germain, 2009). In addition to the genomic mechanisms, non-genomic mechanisms of RA have been discussed. Studies have reported that RA can activate ERK1/2 kinases (Bruck et al., 2009; Waetzig et al., 2019; Cañón et al., 2004) which may have a role in cytoskeletal rearrangement and neurite outgrowth (Pan et al., 2005). Also, retinoylation (RA acylation) - a post-translational alteration of proteins by eukaryotic cells (Das et al., 2014) which may have a role in cell differentiation (Takahashi and Breitman, 1989).

### 5.1 Mechanisms of action

RA can act on many different cells of both the innate and adaptive immune systems (Oliveira et al., 2018). For example, RA may induce proinflammatory cytokines production by DCs, which promotes the differentiation of effector T cells (Hall et al., 2011a). In natural killer (NK) cells, RA suppresses the human NK cell cytotoxicity activated by IFN-α (Abb et al., 1982). RA is essential for B cell production of IgA antibodies playing a multifactorial role in mucosal immunity (Mora et al., 2006). ATRA also inhibits the production of the proinflammatory cytokines TNF-α and IL-12 and potentiates IL-10 production in the THP-1 monocyte cell line and human cord blood mononuclear cells (CBMCs) (Wang et al., 2007). The effects of ATRA on T cells seem to be dependent on the cytokine milieu. In the steady state ATRA functions, in combination with TGFβ, to maintain homeostasis and tolerance through the induction of regulatory T-cells (T-regs). However, in a pro-inflammatory microenvironment ATRA can tip the balance in favour of Th17 and Th1 or Th2 T cell responses (Erkelens and Mebius, 2017; Hall et al., 2011b). In this section, we will discuss the effects that ATRA exerts on macrophages during TB infection.

### 5.2 Promoting autophagy

It has been previously reported that ATRA promotes autophagy in promyelocytic leukaemic cells (Trocoli et al., 2011). Mtb infection inhibits autophagic flux in human macrophages (Petruccioli et al., 2012). ATRA also promotes autophagy in human macrophages infected with Mtb, which results in increased bacterial clearance, by enhancing colocalisation of Mtb with autophagic vesicles and acidified lysosomes. The same effect was reported when ATRA precursors retinol and retinal were used, which was due to their metabolism into ATRA (Coleman et al., 2018). The normal level or serum retinol is 0.7–2.8 μmol per liter (Michaëlsson et al., 2009). Exposure to ATRA (5 μM) in the absence of macrophages had no effect on Mtb growth in comparison to control, which indicates that the antimycobacterial effect of ATRA is based on the signalling within the cell and it is not directly toxic to Mtb at this concentration (Coleman et al., 2018). However higher doses of ATRA (13.3 μM, 4 μg/mL) can directly inhibit growth of mycobacteria (Greenstein et al., 2014).

**Fig. 2:**
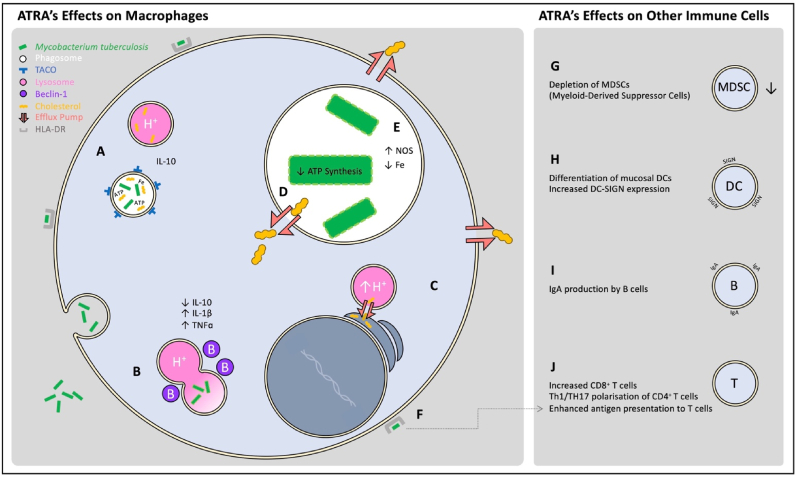
ATRA's mechanisms of action as a HDT against Mtb. (A) Mtb is phagocytosed by macrophages and contained in phagosomes but survives by utilising host-derived cholesterol and iron for its metabolism and inhibiting fusion with lysosomes. (B) ATRA increases macrophage expression of a key pro-autophagy protein, Beclin-1. ATRA also reduces the expression of tryptophan–aspartate containing coat protein (TACO) and IL-10 and increases the expression of cytokines IL-1β and TNF⍺. These factors enable phagolysosomal maturation. (C) ATRA upregulates macrophage expression of multiple cholesterol efflux pumps, such as NPC2, ABCA1 and ABCG1. NPC2 shuttles cholesterol from lysosomes to the endoplasmic reticulum, increasing lysosomal acidity. (D) ABCA1 and ABCG1 reduce intracytoplasmic and intraphagosomal cholesterol concentrations. This starves Mtb of its fuel source, reducing its ability to synthesise ATP. (E) ATRA reduces macrophage intracellular iron concentrations, reducing the availability of a key Mtb nutrient, and increases cellular NOS production. (F) ATRA increases macrophage HLA-DR expression, increasing their ability to present Mtb antigens to T helper cells. (G) ATRA induces maturation and functional depletion of regulatory MDSCs. (H) ATRA increases dendritic cell expression of DC-SIGN, augmenting their antigen-presentation ability. (I) ATRA leads to isotype switching of IgA antibodies in B cells. (J) ATRA increases the counts of CD4^+^ and CD8^+^ T cells *in vivo* and regulates CD4^+^ polarisiation.

TANK-binding kinase 1 (TBK1), is important for autophagic clearance of Mtb, it can regulate type I interferon response induced by dsDNA (Pilli et al., 2012). The STING/TBK1/IRF3 pathway is activated by Mtb via cytosolic sensing of its DNA (Manzanillo et al., 2012). ATRA's antimycobacterial effect is autophagy dependent and inhibition of autophagy, by blocking TBK1, prevented killing of Mtb (Saitoh et al., 2009). Blocking the canonical autophagy pathway resulted in reduced co-localisation of Mtb bacilli with lysosomes and thus reduced the antimycobacterial ability of ATRA. The data indicates that ATRA enhanced the ability of human macrophages to kill Mtb by autophagy and is dependent on PI3 kinase and Beclin-1 (Coleman et al., 2018). Interestingly, the vaccine strain of *M. bovis,* BCG, was found to be resistant to killing by ATRA (Coleman et al., 2018). Mtb expresses the ESX1 secretory system which secretes ESAT-6 allowing bacterial dsDNA to escape from phagosomes and be detected by cytosolic DNA sensors whereas BCG lacks ESX1 and thus remains undetected (Davenne and McShane, 2016). (Fig. 2) (Table 1).

**Table 1:** *In vitro* and *in vivo* studies of ATRA as a HDT against *Mtb*.

	Author	ATRA Dose (Route)	Mycobacterial Strain Used	Cell Type or Animal	Findings	Ref
*In Vitro*	Coleman et al.	ATRA 5 × 10^−6^ M	H37Ra BCG	Murine BMDM Human MDM	Promotion of autophagy Reduced Mtb burden	23
	Crowle et al.	ATRA 1 × 10^−5^ M	Mtb Erdman (ATCC 35801)	Human MDM	Reduced Mtb burden	131
	Wheelwright et al.	ATRA 1 × 10^−8^ M	H37Ra H37Rv	Human monocytes Human MDM	Reduced intracellular cholesterol Reduced Mtb burden	169
	Babunovic et al.	ATRA 1, 10 × 10^−6^ M	H37Rv	Murine BMDM, THP-1, Human MDM	Reduced intracellular cholesterol Reduced Mtb burden	171
	Estrella et al.	ATRA 1 × 10^−6^ M with vitamin D_3_	H37Ra H37Rv	THP-1	Increased antigen presentation Promotion of autophagy Increased ROS production Reduced Mtb burden	173
	Anand et al.	ATRA 0.5 × 10^−6^ M with chenodeoxycholic acid	H37Rv	THP-1	Promotion of autophagy Reduced Mtb burden	177
	Abd-Nikfargam et al.	13-cis-RA 500–2000 × 10^−6^ M	H37Ra	U937	Increased antigen presentation Increased NO production Reduced Mtb burden	184
	O'Connor et al.	ATRA 17 × 10^−6^ M	H37Ra	THP-1	Reduced IL-10 Reduced Mtb burden	174
*In Vivo*	O'Connor et al.	ATRA 2.5 mg/kg (intratracheal)	H37Rv	BALB/c mice	Reduced pathology Reduced Mtb burden	174
	Yamada et al.	ATRA 1 g/kg (oral)	H37Rv	LEW/CrlCrlj rats	Reduced pathology Reduced Mtb burden Increased CD4, CD8 T cells Increased TNFɑ, IL-1β	193
	Mourik et al.	ATRA 2 mg/kg (subcutaneous) with vitamin D_3_ and α-Galactosylceramide	H37Rv	BALB/c mice	Reduced Mtb burden Reduced relapse Increased CD8 T cells, Reduced MDSCs Increased TNFɑ	199
	Knaul et al.	ATRA 5 mg pellet (subcutaneous)	H37Rv BCG	C57BL/6 mice 129S2 mice	Reduced MDSCs Reduced Mtb burden Increased CD4, CD8 T cells	209
	Riccomi et al.	ATRA 300 μg (oral) with subunit vaccine	H37Rv BCG	CB6F1 mice	Increased CD4 T cells Increased mucosal IgA Increased IFNγ, IL-17 Limited later inflammation	224

### 5.3 Reducing intracellular cholesterol and inducing lysosomal acidification

Host-derived lipids (cholesterol and fatty acids) play an important role in the interaction between immune cells and Mtb (Brzostek et al., 2009; KM et al., 2018). In caseous TB granuloma, genes that are involved in lipid metabolism and cholesterol accumulation are upregulated (Kim et al., 2010). Moreover, the presence of foamy macrophages in granulomata and host hypercholesterolemia are correlated with poor protection against Mtb (Martens et al., 2008; Gatfield and Pieters, 2000). Persistence of Mtb within macrophages is dependent on cholesterol – an important nutrient for the bacteria (Griffin et al., 2011, 2012). Moreover, Mtb utilises host lipids to reduce metabolic stress which affects pathogen virulence and immunogenicity (Koo et al., 2012; Lee et al., 2013). Accumulation of lipids in lysosomes changes the lysosome microenvironment by altering its pH to favour Mtb survival (Cox et al., 2007).

ATRA is known to induce cholesterol efflux in macrophages (Costet et al., 2003; Escher et al., 2003). During Mtb infection ATRA reduces the total cellular cholesterol concentration in macrophages, which is thought to contribute to its antimicrobial activity (Wheelwright et al., 2014a). Mutations in NPC2, which is a lysosome-to-endoplasmic reticulum lipid transporter, are the cause of Niemann-Pick disease, a lysosomal lipid storage disorder that is characterised by increased intracellular cholesterol accumulation (Newton et al., 2020). NPC2 expression is decreased in caseous TB granuloma and Mtb infected cells. Treatment with ATRA is associated with increased expression of NPC2, which leads to lower cholesterol content and lysosomal acidification in monocytes and MDMs. The loss of NPC2 expression or inhibition of cholesterol efflux, induced cholesterol accumulation inside the cell and ablated ATRA-induced antimicrobial activity (Wheelwright et al., 2014a). Retinoic acid can also signal through PPAR-β-RXR heterodimers and may alter lipid metabolism and glucose homeostasis – variables known to be important in cellular TB host defense (Mora et al., 2008). Also, ATRA shifts the metabolism of LPS-activated MDMs toward glycolysis, leading to the activation of NLRP3 inflammasome which is required for the production of IL-1β (Alatshan et al., 2020). A recent paper used Mtb CRISPR interference screening in a human macrophage infection model to identify the Mtb genes required to survive in ATRA-treated macrophages, and showed that ATRA treatment starves Mtb of cholesterol and its downstream metabolite propionyl coenzyme A. ATRA did this in part by upregulating macrophage expression of ABCA1 and ABCG1, two cholesterol efflux pumps that have previously been shown to be upregulated in human MDMs by ATRA (Costet et al., 2003; Babunovic et al., 2022). (Fig. 2) (Table 1).

### 5.4 Synergy between ATRA and vitamin D

ATRA and vitamin D are basic components of a healthy diet and have been shown to be linked to a protective immune response against TB (Coleman et al., 2018; Liu et al., 2007). The combination of ATRA and vitamin D has many biological effects on myeloid cells *in vitro* including enhancing the levels of DC-SIGN molecule, which is important for Mtb uptake by human DCs and antigen presentation to T-cells (Tallieux et al., 2003). The combination also increases expression of mannose receptors and decreases Mtb growth by inducing reactive oxygen species (ROS) and autophagy in human THP-1 macrophages (Estrella et al., 2011a). In addition to increased expression of antigen presenting and chemotactic receptors, a multinucleated giant cell (MNGC) phenotype was induced in the THP-1 cells treated with vitamin D and ATRA, in addition to enhanced localisation of Mtb in protease rich lysosomal compartments that hinder bacterial replication was observed (Estrella et al., 2011a). Interestingly, we observed a similar MNGC phenotype in THP-1 cells stimulated with ATRA alone, with or without Mtb infection (O'Connor et al., 2019).

Mtb resides inside a tryptophan aspartate containing coat protein (TACO) -coated stable phagosome that prevents phagosome lysosome fusion in macrophages (Ferrari et al., 1999). Cholesterol mediates the phagosomal association of TACO protein that prevent degradation of Mtb in lysosomes (Gatfield and Pieters, 2000). The synergistic activity of both vitamins have the ability to downregulate the expression of TACO gene in human macrophages (Anand and Kaul, 2003). Another study has revealed that the combination of chenodeoxycholic acid (CDCA) with retinoic acid had the ability to downregulate TACO gene transcription, through FXR/RXR pathway in which the two receptor heterodimerise and cause downregulation of TACO protein and phagolysosomal maturation, which led to poor intracellular survival of Mtb (Anand and Kaul, 2005) (Fig. 2).

### 5.5 Regulation of Nitric oxide (NO) and surface receptors in macrophages

Nitric oxide plays an important role in the control of chronic Mtb infection by stimulating heat-shock protein (HSP) production which starts the stationary phase of Mtb growth (Ryndak et al., 2015; Cunningham-Bussel et al., 2013). The Mtb-infected phagosome has less iNOS activity and reduced respiratory burst capacity (Tomioka et al., 2012; Banerjee and Bhattacharyya, 2014). Macrophages express several receptors that mediate cross-talk with T-cells -such as HLA-DR which plays a major role in presentation of antigen to helper T cells (Lekkou et al., 2004) - and other receptors that bind Mtb cell wall components, altering cytokine secretion. For example, CD14 which may make macrophages more responsive to chemokines enabling bacterial detection by the immune system (Estrella et al., 2011b). Treatment with 13-cis retinoic acid, the isomer and prodrug of ATRA, led to increased expression of HLA-DR and CD14 in U937 macrophagesinhibited the growth of the attenuated H37Ra strain of Mtb *in vitro* and induced NO generation. It was not determined in this study whether inhibition of Mtb growth was dependent on reactive nitrogen species (Abd-Nikfarjam et al., 2018). (Fig. 2) (Table 1).

### 5.6 Reducing intracellular iron

The Mtb-containing phagosome accumulates cellular iron which favours the growth of the bacteria (Johnson et al., 2010). ATRA downregulates cellular transferrin receptors, therefore reducing the supply of iron in phagosomes (Iturralde et al., 1992). Gene expression of peripheral blood mononuclear cells (PBMCs) taken from cynomolgus macaques post vaccination with BCG and post challenge was compared with gene expression when the animals were naïve. Gene expression data revealed an up-regulation of iron regulatory genes in animals that developed TB and down-regulation of these genes in disease controllers, indicating the ability to successfully withhold iron could be important in TB disease control and lowering intracellular iron can limit the infection (Wareham et al., 2014). (Fig. 2) (Table 1).

### 5.7 Alterations in cytokine expression and immune cell numbers *in vivo*

Retinoic acid has the ability to stimulate both innate and adaptive immune response (Iwata, 2003; Ma et al., 2005). Aging rats fed with marginal vitamin A diet had a low number of peripheral blood mononuclear cells and low cell lytic efficacy of natural killer (NK) cells in addition to changes in the distribution and function of T and B cells compared to rats fed a vitamin A replete diet (Paterson et al., 1987; Dawson et al., 1999; Chen and Ross, 2004). Rats infected with the pathogenic strain of Mtb (H37Rv) and treated with ATRA orally were found to have smaller lung granuloma compared with the untreated group (Yamada et al., 2007). Significant increases in the counts of CD4^+^, CD8^+^ T cells, α/β T cells, CD25^+^ T cells, and CD163-positive monocyte/macrophages were observed in rats treated with ATRA (Yamada et al., 2007).

Cytokines play critical roles in the host defense mechanism against TB (Yamada et al., 2000; Sugawara et al., 1999; S et al., 2001) and ATRA can influence their expression. In a 2D *in vitro* model of Mtb infection, ATRA inhibited IL-10 secretion by THP1 macrophages (O'Connor et al., 2019), which may allow phagolysosomal maturation to proceed, as previously reported (O'Leary et al., 2011). Retinoic acid has a role in the regulation of IFN-γ signalling by regulation of several components of the IFN-γ signalling pathway (Luo and Ross, 2005). The mRNA levels of IL-1β, TNFα, and iNOS mRNA expression were elevated in the lung tissues of rats with TB treated orally with RA and in *in vitro* treated bronchoalveolar lavage (BAL) cells (Yamada et al., 2007). A TB mouse model treated with ATRA, vitamin D3, and alpha-galactosylceramide plus the standard antibiotics showed increased TNF-α protein levels in the lungs during the treatment course in addition to increased CD8^+^ cells, compared to antibiotics alone (Mourik et al., 2017). Our *in vivo* evaluation of ATRA loaded Poly lactic-*co*-glycolic acid (PLGA) microparticles and free ATRA (2.5 mg/kg of ATRA) delivered locally to the lungs of BALB/c mice infected with H37Rv Mtb strain, demonstrated reduced bacterial burden in comparison to controls, both as a standalone or adjunctive to rifampicin. In addition, treatments reduced both lesion size and pulmonary pathology in this model (O'Connor et al., 2019), which might reflect the simultaneously anti- and pro-inflammatory effects of ATRA. One possible explanation of reduced inflammation following ATRA treatment is the expansion of regulatory T-cells in the lungs (Coleman et al., 2013). (Fig. 2) (Table 1).

### 5.8 Depleting myeloid suppressor cells

Myeloid-derived suppressor cells (MDSCs) are a diverse population of myeloid origin that negatively regulate immune function (Garg, 2021). In cancer, MDSCs have been shown to regulate immunity at the tumour site (Haverkamp et al., 2011). Research done in animal models, has demonstrated the inhibition of antimicrobial activity by MDSC (Garg et al., 2017; Delano et al., 2007). Elevated suppressive myeloid cells in blood and pleural effusions of TB patients have been reported in active TB patients (Du Plessis et al., 2013). Immune regulation through MDSCs is multifunctional and includes deprivation of environmental nutrients necessary for T-cell function, induction of regulatory T-cells or IL-10 secretion and inhibition of interferon gamma (IFN-γ) production (Gabrilovich et al., 2012; Gabrilovich and Nagaraj, 2009). Th1 cytokines are required for fighting the infection but can also lead to excessive inflammation and tissue damage; the interaction between T lymphocyte and myeloid cells may provide a delicate balance for disease control (O'Garra et al., 2013).

Targeted inhibition of MDSCs may contribute to successful anti-TB treatment. ATRA was shown to induce maturation and functional depletion of MDSCs (Mora et al., 2008) and decreases MDSCs frequency (Leukes et al., 2021). Knaul et al., found that in murine model of TB, MDSCs accumulate and reside in lung parenchyma providing a niche for Mtb propagation. ATRA treatment decreased lung MDSCs, increased T-cell numbers and diminished the capacity of mycobacteria infected bone marrow derived MDSCs to suppress T-cell proliferation without affecting cytokine responses or cell death in their model (Knaul et al., 2014). Targeting MDSCs using ATRA may be beneficial in treating patients with drug resistant TB and the elderly with elevated MDSCs (Verschoor et al., 2013). The combination of ATRA and alpha-galactosylceramide has been found to convert MDSCs into immunogenic antigen-presenting cells (Lee et al., 2012). Data has shown that ATRA increased CD1d expression on antigen-presenting cells, which is required for activation of natural killer T (NKT) cells (Chen et al., 2013). Moreover, the addition of immunotherapy consisting of the clinically approved drugs all *trans* retinoic acid, 1,25(OH)2-vitamin D3, and a-galactosylceramide to the standard antibiotic treatment reduced bacterial load in the lungs after 5 weeks of treatment and reduced relapse of disease at 13 weeks post treatment course which was accompanied by decreased numbers of MDSCs in a mouse model of TB (Mourik et al., 2017). To what extent the beneficial effects of ATRA on TB pathogenesis in the above-mentioned studies (Paterson et al., 1987; Banerjee and Bhattacharyya, 2014) are due to depletion of MDSCs and/or the ability of ATRA to boost macrophage microbicidal activity remains to be determined (Fig. 2) (Table 1).

### 5.9 ATRA as a vaccine adjuvant

Most microbes including Mtb invade the human body through mucosal surfaces and thus, strengthening mucosal immunity is a pivotal factor in host defence against those microbes (Hellfritzsch and Scherlieβ, 2019). Mucosal immunity elicits effective humoral and cellular immune responses both at the mucosa and systematically (Srivastava et al., 2015). However, there is an urgent need to develop safe and effective vaccine adjuvants to induce a mucosal immune response.

Retinoic acid (RA) has been proven to induce immune modulation at mucosal sites (Erkelens and Mebius, 2017). It controls DCs homeostasis at mucosal sites (Evans and Reeves, 2013) and regulates differentiation of CD4^+^ T-cells toward Th1/Th17 polarisation (Hall et al., 2011b), which is required for an effective response in the early stages of Mtb infection. Antigen presentation in presence of retinoic acid (RA) confers a mucosal homing phenotype on B and T cells (Mora et al., 2006; Iwata et al., 2004). The effect of RA on B cells leads to isotype switching of IgA antibodies (Lee et al., 2016) and it induces homing of antigen specific T cells in mucosal surfaces including the lungs (Tan et al., 2011). In the absence of RA-mediated signalling, defective T-cell differentiation occurs at the mucosa and other tissues (Surman et al., 2014; Kaufman et al., 2011). RA is essential for the differentiation of mucosal DCs (Cassani et al., 2012). These effects could explain why people with VAD are more susceptible to infection as previously mentioned.

It has been proposed that immunity against Mtb infection is linked with T cells homing to the lungs (Sakai et al., 2014; Woodworth et al., 2017). The subcutaneous vaccination of mice with the subunit vaccine CAF01+H56 in the presence of RA caused increased pro-inflammatory cytokine secretion and homing of mucosal H56 specific IgA and Mtb specific CD4^+^ T-cells to the lungs in comparison with mice vaccinated in the absence of RA. Although the effect of RA was transient, the host was able to better contain the inflammatory response and more Mtb specific CD4+PD1+ T-cells were found at later time points which limited host damage (Riccomi et al., 2019). Therefore, more attention should be given to studying the effect of RA as a mucosal vaccine adjuvant in infectious disease (Table 1).

## 6 Clinical trials of vitamin A for TB and why they have failed

### 6.1 Clinical trials of vitamin A for TB

No human clinical trials evaluating ATRA as a TB HDT have been undertaken. Only one relevant case report has been published, which describes a patient prescribed ATRA for acute promyelocytic leukaemia in addition to Mtb therapy, and who developed hypercalcemia as a possible adverse effect of ATRA (Abdullah et al., 2018). However, many trials evaluating a range of oral doses of retinol supplementation (typically 5000 IU daily or 200,000 IU as a single dose), as esters, as an adjunct to TB therapy have taken place. These trials in Mexico (Armijos et al., 2010), Nigeria (Lawson et al., 2010a), Indonesia (Karyadi et al., 2002; Pakasi et al., 2010), South Africa (Visser et al., 2011; Hanekom et al., 1997), Malawi (Semba, 1999b), China (Wang et al., 2020), and India (Ginawi, 2021) have included both adult and pediatric (HIV-seropositive and HIV-seronegative) patients. While some trials have indicated a trend towards earlier sputum conversion in the retinol-supplemented group (Armijos et al., 2010; Lawson et al., 2010a; Karyadi et al., 2002; Pakasi et al., 2010; Visser et al., 2011), a 2016 meta-analysis found that overall, oral retinol supplementation did not significantly affect sputum positivity at 2 weeks, 1 month or 2 months. No significant difference in mortality was found (Grobler et al., 2016). No trials of retinol or ATRA prescribed for the prevention of active TB in contacts, LTBI or other high-risk groups have been undertaken.

### 6.2 Why clinical trials have failed

**Fig. 3:**
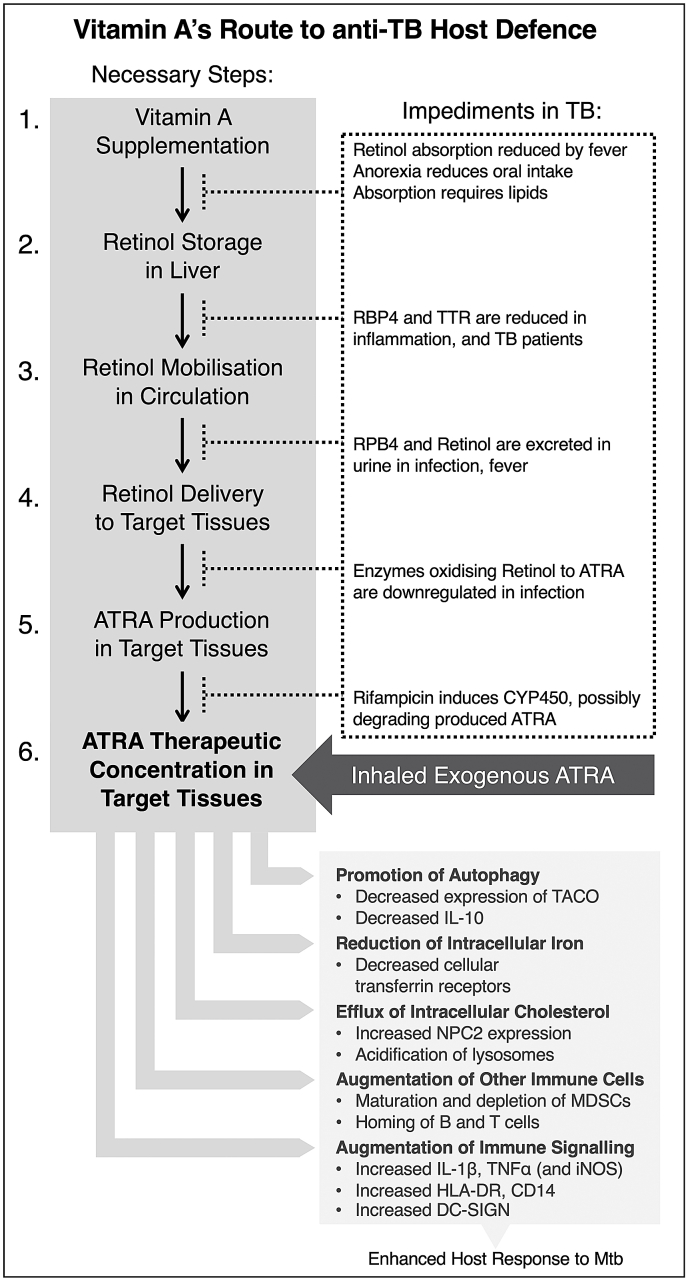
ATRA has pleiotropic effects that protect the host against Mtb (lower-right box). These include: The co-localisation of Mtb with autophagic vesicles and promotion of phagolysosomal maturation through a reduction in the expression of TACO and a reduction in IL-10; A reduction in the cellular expression of transferrin receptors, reducing the supply of a vital nutrient of Mtb – iron; The efflux of another Mtb nutrient, cholesterol, and the acidification of lysosomes via increased NPC2 expression; A functional depletion of MDSCs and improvement of the homing capacity of B and T cells; And the augmentation of innate immune signalling, characterised by an increase in HLA-DR, CD14, DC-SIGN and pro-inflammatory cytokine secretion. There are many necessary steps that dietary vitamin A must undertake in order to exert these host-protective effects (upper-left box). First, supplemented retinoids must be absorbed by enterocytes, secreted in chylomicrons via the lymphatics into circulation and endocytosed by the liver for processing and storage. Retinol must then be secreted with its carrier proteins into circulation and delivered to its target tissues. Once intracellular, retinol must be converted to its active metabolites in a two-step oxidation reaction before it can be transported to its nuclear receptors. However, TB patients present many obstacles to these steps (upper-right box). For example, retinoid absorption can be reduced by fever, anorexia, or a lipid-poor diet. Retinol's circulatory carrier proteins (RBP4 and TTR) are negative acute phase proteins that are suppressed in TB patients. Retinol is excreted from the circulation in urine during fever and infection. The macrophages of TB patients may not be capable of producing ATRA from retinol, and anti-tuberculous medications may increase the degradation of ATRA that is locally produced. Many of these obstacles might be bypassed by using inhaled exogenous ATRA as a HDT.

Both retinol and ATRA enhance the Mtb-killing ability of human macrophages *in vitro* (Coleman et al., 2013; Crowle and Ross, 1989; Anand and Kaul, 2005; Wheelwright et al., 2014b; Long et al., 2016; da Cunha et al., 2014), and are suggested to hold promise as a HDT for Mtb (O'Connor et al., 2016). There are several possible explanations for why retinol has failed to improve outcomes in clinical trials in Mtb infection (Fig. 3):

1. Absorption of oral retinol supplementation is negatively correlated with fever (Aklamati et al., 2010), and requires a diet containing lipids.
2. Baseline liver vitamin A reserves of TB patients may be severely depleted by the subacute Mtb infection itself, the associated anorexia, other infections and malnutrition (Stephensen, 2001). This is supported by the low baseline retinol levels, low BMI and the lack of difference in retinol trajectory between intervention and placebo arms observed in many of the trials (Armijos et al., 2010; Lawson et al., 2010a; Karyadi et al., 2002; Pakasi et al., 2010; Visser et al., 2011; Hanekom et al., 1997; Semba, 1999b; Wang et al., 2020).
3. Infection and fever are strongly associated with urinary retinol excretion (Alvarez et al., 1995; Stephensen et al., 1994). This may counteract supplementation efforts.
4. It is possible that in practice, active TB patients present at too late a stage, and that retinol would be most effective at enhancing early clearance of the bacillus.
5. The serum carrier proteins that transport retinol to its sites of action, RBP4 and TTR, are both significantly reduced in TB patients (Keicho et al., 2012; Agranoff et al., 2006). RBP4 is a negative acute phase reactant that declines with inflammation (Larson et al., 2017; Louw et al., 1992). Baseline C-Reactive Protein (CRP) and Erythrocyte Sedimentation Rate (ESR) were elevated among trial patients (Lawson et al., 2010a; Karyadi et al., 2002; Pakasi et al., 2010; Visser et al., 2011; Wang et al., 2020).
6. Rifampicin is a potent CYP450 enzyme-inducer. As has been shown for other CYP450-inducers, rifampicin may significantly increase clearance of ATRA and thereby reduce retinol's effectiveness (Fex et al., 1995; Nau et al., 1995).
7. Inflammation downregulates murine intestinal macrophage expression of ALDH enzymes which are required for the production of ATRA from retinol (Hurst and Else, 2013). In addition, ALDH1a2 and DHRS9 expression are reduced in human TB granulomata (Kim et al., 2019). The macrophages of TB patients may not be capable of effectively producing ATRA from retinol.
8. ATRA is promising as a TB HDT as it both enhances autophagy and contributes to immune regulation, reducing unwanted tissue damage. Nevertheless, specific HDTs may be effective only in patients with specific TB phenotypes or endotypes (DiNardo et al., 2020, 2021).

Two compatible strategies might sidestep several of these obstacles. The first is to use ATRA rather than retinol as an adjunct to TB-therapy. This would remove the need for retinol absorption, storage, mobilisation and the intracellular oxidation of retinol to ATRA. The second is to target the lung with ATRA directly rather than systemically by using an inhaled formulation. This could increase the concentrations of ATRA at the site of interest, while reducing systemic effects. Randomised controlled trials of ATRA as an adjunct to TB therapy could first be undertaken in patients failing TB therapy with extensively drug-resistant TB (Fig. 3).

## 7 Potential of vitamin A as a HDT in other infectious diseases

### 7.1 Animal studies

Retinoic Acid could prove to be an exciting therapy in other infections, as demonstrated by promising results from animal studies. ATRA was investigated as a HDT for the opportunistic fungal pathogen, *Pneumocystis jiroveci* in immunosuppressed mice and rats. The rodents were commenced on ATRA orally 3 weeks post-inoculation with *Pneumocystis*. It was observed that ATRA treatment could cure pneumocystis pneumonia and greatly reduced lung inflammation, but that it required a prolonged course of therapy. Combining ATRA with primaquine was as effective as the more toxic first-line therapy, co-trimoxazole (Lei et al., 2013). Another animal study found that intraperitoneal ATRA pre-treatment greatly improved the survival of BALB/c mice when challenged with an intravenous infection of the intracellular gram-positive rod, *Listeria monocytogenes*. By day 5 post-infection, none of the control mice but 66.6% of the ATRA-treated mice survived. It was found that ATRA-treated mice had decreased circulating pro-inflammatory cytokines and reduced visceral burdens of bacilli. The authors also found that ATRA improved the phagocytosis of *L. monocytogenes* by J774 cells *in vitro* (Castillo et al., 2015).

### 7.2 Human clinical trials

While ATRA has not been trialed in other infections, several trials have been registered that propose to investigate 13-*cis*-RA as a treatment for COVID-19 infection (NCT04353180, NCT04361422, and NCT04663906), which are currently in pre-recruitment stages. Two clinical trials of oral retinol as an adjunctive therapy for pneumonia in pediatric patients were undertaken in the 1990s. The trials in Guatemala and Peru did not observe a clinical benefit to retinol treatment (Kjolhede et al., 1995; Stephensen et al., 1998). Retinol has also been proposed as a treatment worth investigating for COVID-19 infection in low-resource settings (Midha et al., 2020).

### 7.3 ATRA for non-infectious diseases

In addition to ATRA's established efficacy in the treatment of acute promyelocytic leukaemia, its potential use in other infections and 13-*cis*-RA's established efficacy in the treatment of acne vulgaris, ATRA (given orally in capsule formulation) has also been trialed as a treatment in several non-infectious diseases. These include emphysema (Mao et al., 2002; Roth et al., 2006), a phase 1 study in patients with solid tumors (Conley et al., 1997), a phase 1 study in pancreatic cancer (Kocher et al., 2020), a phase 2 study in metastatic breast cancer (Sutton et al., 1997) and a phase 2 study in prostate cancer (Trump et al., 1997). These studies have established ATRA's pharmacokinetics and pharmacodynamics properties, as well as its toxicities in a range of patients. A study of ATRA treatment in patients with renal cell carcinoma found that ATRA had no effect on the total white cell count, neutrophil count or lymphocyte count, but did result in a mild, transient reduction in monocyte count (Mirza et al., 2006). While ATRA is generally well tolerated, the adverse effects most frequently observed are headache, xeroderma, hypertriglyceridemia and cough. RA is also teratogenic and contraindicated in pregnancy (Rothman et al., 1010), limiting its potential use as a TB HDT in this population.

## 8 Formulation and delivery strategies for ATRA

Some studies have showed that vitamin A supplementation itself has no added value in TB treatment (Lawson et al., 2010a, 2010b). These findings could be due to the complex *in vivo* metabolism of vitamin A to ATRA as previously discussed. ATRA displays poor aqueous solubility and reduced half-life in plasma (Szuts and Harosi, 1991; Muindi et al., 1992). At higher concentrations, it can cause toxicity and may not reach the target cells in the desired concentration. Therefore, administration of ATRA locally to achieve high concentration at the site of infection is important to avoid systematic side effects (Gonçalves et al., 2019; Guerra et al., 2014).

Vitamin A is a fat soluble vitamin which requires a formulation step before *in vivo* administration (Huyghebaert et al., 2007). Free ATRA cannot be easily aerosolised due to its lipophilicity and inherent instability during manufacturing and storage. Vitamins are susceptible to degradation when exposed to air, heat, light, moisture and certain pHs (Estevinho et al., 2016). Loading ATRA into nano/microcapsules has been demonstrated to improve its performance by minimizing side effects and increasing stability and half-life (Gonçalves et al., 2019). Several topical lipid based formulations encapsulating retinoic acid have been studied for treatment of acne including emulsions, solid lipid nanoparticles (SLN), nanostructured lipid carriers (NLC) liposomes, niosomes and ethosomes which are known for their biocompatibility and sustained release profile (Lin et al., 2013; Silva et al., 2015; Raza et al., 2013). In humans, A phase I/II clinical trial of intravenous (IV) ATRA-loaded liposomes for renal cell carcinoma treatment has been reported (Boorjian et al., 2007). Polymeric nanocarriers have also been studied for RA encapsulation including inhaled poly-lactic-*co*-glycolic acid (PLGA) microparticles for TB (O'Connor et al., 2019) and loading ATRA in styrene maleic acid copolymer (Yamamoto et al., 2013). ATRA has also been incorporated into collagen-hyaluronate for respiratory tissue generation (O’Leary et al., 2016, 2017). The drug can be formulated in multi-drug formulations with another first line anti-TB drug in order to reduce the patient's oral medication burden (O'Connor et al., 2019).

Mtb is transmitted by inhalation of contaminated respiratory droplets and the lungs are the main site of infection (Pai et al., 2016). Pulmonary drug delivery directly to the site of infection leads to less systematic toxicity than oral or parenteral delivery and represents a promising route to deliver drugs directly to the lungs (O'Connor et al., 2019; Wauthoz et al., 2011). Drug delivery by inhalation has been used since ancient Egyptian times (Sanders, 2007). Despite that, no inhaled TB treatment has made it to the market yet. It is worth noting that in 2018, the FDA approved the first nebulised amikacin liposome inhalation suspension for *Mycobacterium avium* complex (MAC) (Hoy, 2021). A phase I clinical trial of capreomycin dry powder formulation for drug resistant TB, demonstrated safety in healthy adult volunteers (Dharmadhikari et al., 2013). A study examining the delivery of rifampicin via inhalation in pigs found a 7–9 fold increase in concentration of rifampicin in the lungs compared to delivery via other routes (Garcia Contreras et al., 2015). Delivering ATRA by inhalation may increase its potential as a HDT.

Therefore, administration of ATRA locally to achieve high concentration at the site of TB infection is important to avoid systematic side effects and improve treatment efficacy (Gonçalves et al., 2019; Guerra et al., 2014). Inhaled ATRA-loaded liposomes were safely administered by inhalation in an emphysema patient (Frankenberger et al., 2009). Desai et al. prepared ATRA loaded niosomes and evaluated their aerosol properties; their results showed good encapsulation efficiency and an aerosolised droplet size suitable for inhalation for lung cancer treatment (Desai and Finlay, 2002). Our lab has previously prepared PLGA microparticles of ATRA and this formulation retained antibacterial efficacy and reduced pulmonary pathology compared to ATRA solution in a mouse model of Mtb (O'Connor et al., 2019).

In addition to the effect of the cargo, the carrier itself may have a role influencing immune cell function (Lawlor et al., 2016). In our study, treatment with inhaled ATRA encapsulated in PLGA microparticles led to reduced transcription of TNF-α and iNOS in the lungs of Balb/c mice infected with H37Rv in comparison to ATRA alone (O'Connor et al., 2019). The reduction of iNOS is linked to improved disease pathology in an acute lung injury model (Zhang et al., 2016). Encapsulation of ATRA into drug delivery systems provides the protection and stability required for retinoids. Moreover, this strategy provides a controlled drug release profile and increases the bioavailability of retinoids in the human body. There are several patents of ATRA loaded polymeric nanoparticles (20160338984A1 - Particl, 2016) and liposomes (Composite of all-trans-re, 2010) including ATRA-loaded liposomal aerosols for delivery to the lungs (Liposomal aerosols for de, 2002). Several strategies have been studied to increase particle uptake, by actively targeting the alveolar macrophages, including decoration of the particle surface with mannose (Hatami et al., 2019), glucose (Dube et al., 2014) surfactant protein D and A (Kendall et al., 2013; Ruge et al., 2016).

For any molecule to be delivered via the inhaled route, a suitable device is critical. The key role for the devices is to enable aerosolisation of particles or droplets effectively by generating optimum aerodynamic diameter is 1–5 μm in order to prevent particle exhalation or throat impaction (Hickey et al., 2013; Parumasivam et al., 2016). The main types of medical devices for inhaled therapies include: 1) pressurised metered dose inhaler (pMDI) (Misra et al., 2011). 2) Dry powder inhalers (DPI) (Misra et al., 2011). 3) Nebulisers, which can be divided into three main types depending on the mechanism of aerosol generation: jet, ultrasonic and vibrating mesh nebulisers (Misra et al., 2011). However, nebulisers require a power supply and continuous cleaning of the device, which might limit its use and making DPIs a suitable choice for developing countries where TB is prevalent.

## 9 Concluding remarks

The effect of ATRA on Mtb occurs via an indirect action on the bacteria. More investigation is needed to uncover how ATRA and other retinoids boost the microbicidal activity of macrophages and other immune cells. Future animal studies, examining models of both early and chronic TB infection, should consider measurement of the effects of ATRA on chemokine and cytokines levels, histopathology, cell recruitment and antigen presentation in addition to its effects on bacterial viability. Later experimental time points should be considered as the benefit of a HDT may be delayed when compared with pathogen-directed therapies such as rifampicin. Pharmacokinetic studies comparing the inhaled with other possible routes of administration, and studying ATRA in combination with conventional TB therapies, are also essential. Careful dose-selection for clinical trials is necessary, as the ATRA dose effective in TB may not be directly comparable to that used for other indications. Less toxic synthetic ATRA analogues should also be investigated for their effects on intracellular Mtb growth. Trials evaluating the efficacy of retinoids for the prevention of active TB in high-risk groups should be considered. As has occurred in patients with emphysema and several malignancies, pilot trials of adjunctive ATRA in patients on TB treatment should be pursued. Detailed phenotypic and endotypic characterisation of these trial patients may be critical in understanding and identifying with precision which patients might benefit from this promising HDT.

## CRediT authorship contribution statement

**Ahmad Z. Bahlool:** Investigation, Writing – original draft, Writing – review & editing. **Conor Grant:** Investigation, Writing – original draft, Writing – review & editing, Visualization. **Sally-Ann Cryan:** Conceptualization, Writing – review & editing, Supervision. **Joseph Keane:** Writing – review & editing, Supervision. **Mary P. O'Sullivan:** Conceptualization, Writing – review & editing, Supervision.

## Declaration of competing interest

The authors declare that they have no known competing financial interests or personal relationships that could have appeared to influence the work reported in this paper.
